# Comparison of active measurements, lichen biomonitoring, and passive sampling for atmospheric mercury monitoring

**DOI:** 10.1007/s11356-024-33582-6

**Published:** 2024-05-14

**Authors:** Jan Gačnik, Igor Živković, Jože Kotnik, Dominik Božič, Antonella Tassone, Attilio Naccarato, Nicola Pirrone, Francesca Sprovieri, Alexandra Steffen, Milena Horvat

**Affiliations:** 1 Department of Environmental Sciences, Jožef Stefan Institute, Ljubljana, Slovenia; 2 Jožef Stefan International Postgraduate School, Ljubljana, Slovenia; 3 Institute of Atmospheric Pollution Research, National Research Council, Rende, Italy; 4 Department of Chemistry and Chemical Technologies, University of Calabria, Rende, Italy; 5 Air Quality Research Division, Environment and Climate Change Canada, Toronto, Canada

**Keywords:** Atmospheric mercury monitoring, Lichen biomonitoring, Passive sampling, Active measurement, Method comparison

## Abstract

The number of atmospheric mercury (Hg) monitoring stations is growing globally. However, there are still many regions and locations where Hg monitoring is limited or non-existent. Expansion of the atmospheric Hg monitoring network could be facilitated by the use of cost-effective monitoring methods. As such, biomonitoring and passive monitoring offer a unique alternative to well-established monitoring by active measurements, since they do not require a power supply and require minimal workload to operate. The use of biomonitoring (lichens and mosses) and passive air samplers (PASs) (various designs with synthetic materials) has been reported in the literature, and comparisons with active measurement methods have also been made. However, these studies compared either biomonitoring or PASs (not both) to only one type of active measurement. In our work, we used transplanted (7 sampling sites) and in situ lichens (8 sampling sites) for biomonitoring, two PASs from different producers (3 sampling sites), and two different active measurement types (continuous and discontinuous active measurements, 1 and 8 sampling sites, respectively) to evaluate their effectiveness as monitoring methods. In the 9-month sampling campaign, 3 sampling locations with different characteristics (unpolluted, vicinity of a cement plant, and vicinity of a former Hg mine) were used. The results obtained with lichens and PASs clearly distinguished between sampling locations with different Hg concentrations; using both PASs and lichens together increased the confidence of our observations. The present work shows that biomonitoring and passive sampling can be effectively used to identify areas with elevated atmospheric Hg concentrations. The same can be said for discontinuous active measurements; however, the discrepancy between atmospheric Hg concentrations derived from PASs and discontinuous active measurements should be further investigated in the future.

**Graphical Abstract:**

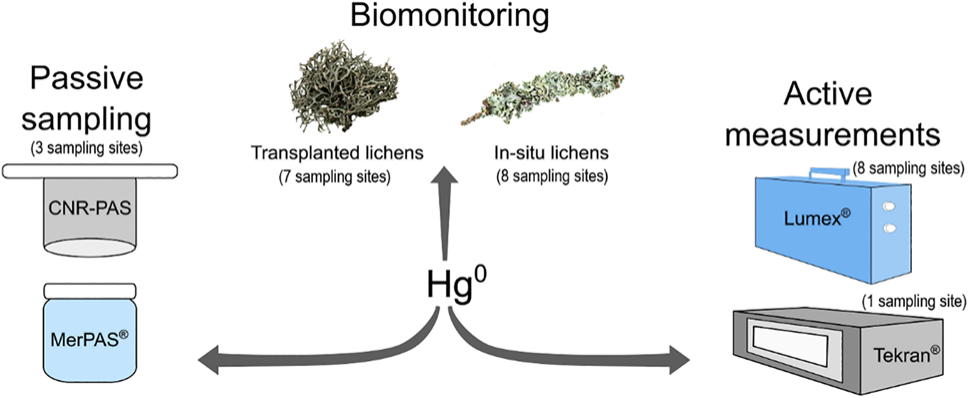

**Supplementary Information:**

The online version contains supplementary material available at 10.1007/s11356-024-33582-6.

## Introduction

Elevated concentrations of Hg in the environment are the result of human activity (Amos et al. 2015) Because most anthropogenic Hg enters the environment through the atmosphere (Driscoll et al. 2013), the chemistry and concentrations of atmospheric Hg are important for understanding human impact on the biogeochemical cycling of Hg. In recent decades, much effort has been invested in improving the knowledge of atmospheric mercury, including the establishment of networks for long-term monitoring of atmospheric Hg. Several such global and regional networks were established in Europe, USA, Canada, and East Asia (Cole et al. 2013; Gay et al. 2013; Sprovieri et al. 2016). Although the number of monitoring sites is increasing, there are still many large regions with limited or non-existent monitoring sites for atmospheric Hg. This is especially true for the Southern Hemisphere (UN Environment 2019).

The majority of monitoring sites use automated continuous measurements to monitor atmospheric Hg concentrations (i.e., by using Tekran units) (Cole et al. 2013; Gay et al. 2013; Sprovieri et al. 2016), although different methods for atmospheric Hg monitoring such as sorbent membranes are increasingly used (Gustin et al. 2021; Luippold et al. 2020). Additionally, a portable atmospheric Hg analyzer can be used for monitoring purposes (Mashyanov et al. 2021; Pandey et al. 2011). The abovementioned monitoring methods can all be considered active measurement methods, requiring a power supply to acquire data. In contrast, biomonitoring and passive samplers (PASs) do not require a power supply and may be cost-effective. For Hg biomonitoring, lichens or mosses are most commonly used (Bargagli 2016). Both in situ and transplanted lichens and mosses can be used for atmospheric Hg monitoring (Boquete et al. 2013; Horvat et al. 2000). PASs are made of synthetic Hg collection materials that can be assembled into different shapes such as radial, axial, and box-shaped PASs. Atmospheric Hg concentration is then derived by knowing the parameters of Hg diffusion to the collection material (Huang et al. 2014).

Atmospheric Hg usually consists mostly of elemental Hg (Hg^0^), though it has recently been shown that oxidized Hg species (Hg^II^) and Hg bound to particulates (Hg-p) could account for about 25% of the total atmospheric Hg concentration (Gustin et al. 2015). Unlike the monitoring of Hg^II^ and Hg-p, which faces analytical challenges and problems, Hg^0^ is monitored routinely with active monitoring methods. On the other hand, biomonitoring and passive sampling methods are not yet routine and well-accepted on a worldwide scale (Bargagli 2016). When using biomonitoring, it is assumed that the accumulation of atmospheric Hg occurs mainly through the uptake of Hg^0^ (Monaci et al. 2022), which can be irreversible due to the transformation to relatively immobile Hg^II^ species or reversible with the desorption of Hg^0^ back into the atmosphere (Lodenius 2013). In most studies using biomonitoring, total Hg content in chosen biota is positively correlated with atmospheric Hg^0^ concentration (Božič et al. 2022; Monaci et al. 2022; Sutton et al. 2014). Correlations between Hg in biota and atmospheric Hg are often highly dependent on parameters such as wind fluxes, air temperature, light, mercury speciation, and biotic species (Lodenius 2013). Due to the dependence on too many factors, biomonitoring methods are most often not calibrated (Huang et al. 2014). On the other hand, PASs are calibrated (McLagan et al. 2018b), also taking into account the effect of meteorological parameters, in particular wind speed and temperature (McLagan et al. 2017). PASs most commonly sample atmospheric Hg^0^ (Cha et al. 2020; Snow et al. 2021; Zhang et al. 2012). Attempts have also been made to introduce PASs which could only sample atmospheric Hg^II^ (Huang and Gustin 2015; Lyman et al. 2010).

Comparative studies of active measurements and biomonitoring/PASs have already been reported in the literature. However, these studies compared only one type of PAS or biomonitoring and/or only one type of active measurement, but not all three monitoring strategies at the same time (Bargagli et al. 2002; McLagan et al. 2018b; Monaci et al. 2022; Naccarato et al. 2021; Sutton et al. 2014). In our present work, we used different biomonitoring methods (transplanted lichens *Punctelia subrudecta* and *Flavoparmelia caperata* and in situ lichens *Hypogymnia physodes*), two PASs developed at the University of Toronto and commercialized by Tekran (MerPAS®) and the Italian National Research Council – Institute of Atmospheric Pollution Research (CNR-PAS), and two active measurement strategies (continuous measurements with a Tekran mercury vapor analyzer and discontinuous measurements with a portable Lumex Hg Analyzer). These monitoring methods were deployed at sampling locations with potentially different atmospheric Hg concentrations. The results were compared and evaluated to assess the suitability of different methods for atmospheric Hg monitoring.

## Experimental

### Sampling locations

**Fig. 1:**
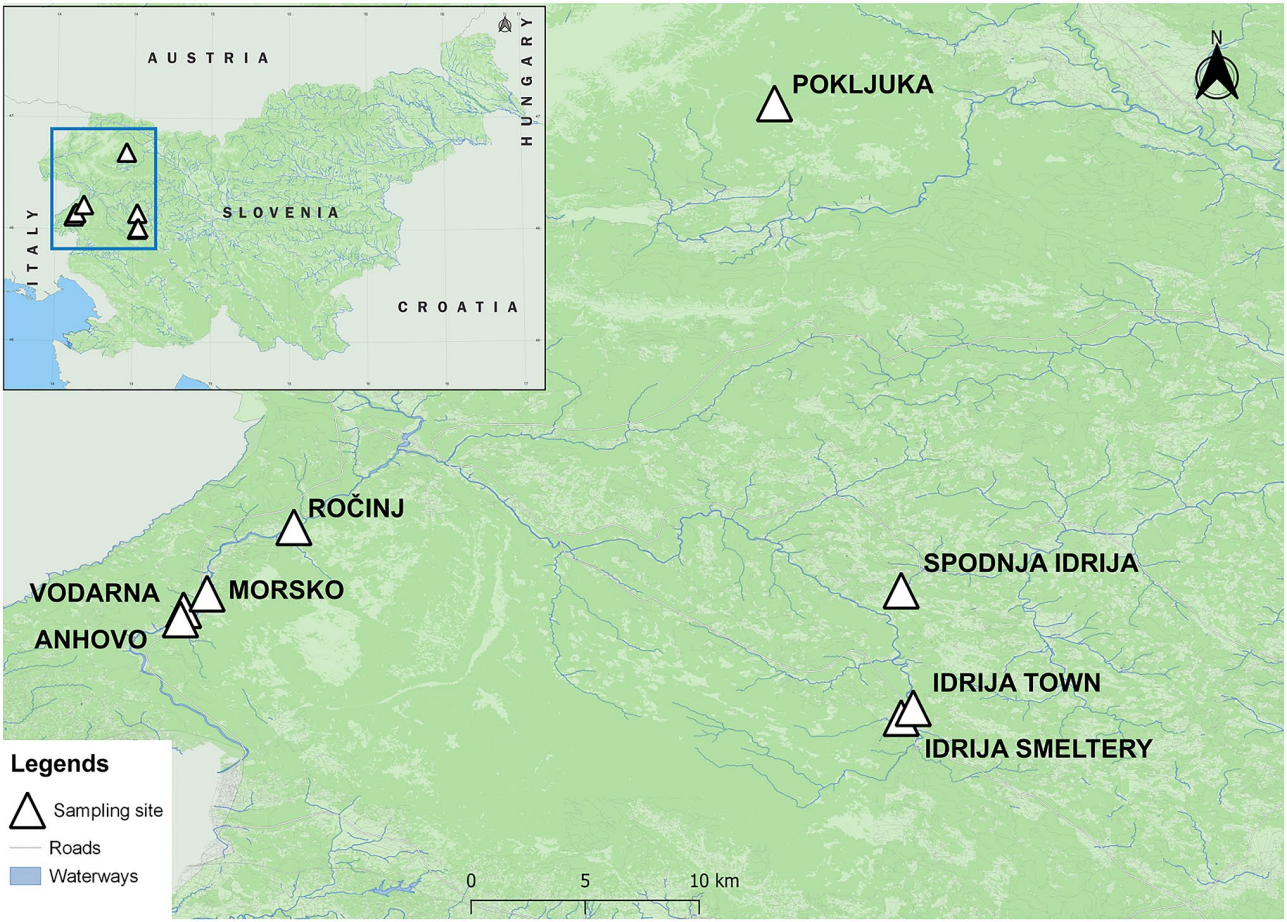
Sampling sites and locations used in the present work

A total of three sampling locations were selected in our work: Anhovo, Idrija, and Pokljuka. Anhovo and Idrija are known for elevated Hg concentrations due to cement production and the former Hg mine, respectively. Location Pokljuka is a remote and clean area, distant from any pollution sources. Four sampling sites were selected in Anhovo (Vodarna, Anhovo, Morsko, and Ročinj), three sampling sites in Idrija (Spodnja Idrija, Idrija Town, and Idrija Smeltery), and one sampling site in Pokljuka. The sampling sites and locations are shown in Fig. 1. The geographic coordinates of each sampling site are shown in Table S1.

Anhovo is a small village in western Slovenia in the narrow Soča Valley. The valley is influenced by the cement factory Salonit Anhovo, which is the largest producer of cement in Slovenia. Mass balances of Hg in the plant cycles are known; about two-thirds of the released Hg is emitted as gaseous Hg^II^ and about one-third as gaseous Hg^0^, whereas Hg-p represents only a small fraction of total Hg (Mlakar et al. 2010). The town of Idrija lies in the Idrija basin, surrounded by the Idrija hills. Mercury was discovered in Idrija in the late fifteenth century; mining and smelting operations lasted there until 1995. During the 500 years of mining history, more than 40,000 tons of Hg were lost to the local environment (Hylander 2002). Pokljuka is a high karst plateau covered with woods in the Julian Alps, one of the most remote areas of Slovenia. It is the largest completely wood-covered surface in the Triglav National Park, more than 20 km wide and almost as long. Pokljuka is considered a remote, clean, pollution-free area where lichens have been collected for transplantation to other sampling sites.

### Sampling methods and analysis

We used active measurements, biomonitoring, and PASs for monitoring Hg in ambient air. Active measurements were done continuously (Tekran speciation system) or discontinuously (Lumex), biomonitoring was done by in situ and transplanted lichens, and passive sampling was done by PASs produced by two different producers (CNR-PAS and MerPAS®). The locations where each method was used and method deployment dates are shown in Table 1. Additional information regarding the deployment plan of transplanted lichens and PASs (start and end of exposure) are available in Table S1 and Table S2 of the Supplementary Information, respectively.

**Table 1:** Locations and sites where active measurements, lichens, and PASs were deployed

Location	Anhovo				Idrija			Pokljuka		
Site	Vodarna	Anhovo	Morsko	Ročinj	Spodnja Idrija	Idrija Town	Idrija Smeltery	Pokljuka		
Continuous active measurements	✓								Deployment period	17/2/2020–13/5/2020
Discontinuous active measurements	✓	✓	✓	✓	✓	✓	✓	✓		16/10/2020–11/11/2020
In situ lichens	✓	✓	✓	✓	✓	✓	✓	✓*		/
Transplanted lichens	✓	✓	✓	✓	✓	✓	✓			16/1/2020–6/5/2020
PASs (all types)	✓	✓				✓				17/2/2020–16/10/2020

^*^Lichens from this site were used for transplantation

The deployment periods did not overlap completely for all deployed methods. This matter is discussed more closely in the results and discussion section for each individual case; in general, we compared methods during overlapping deployment periods if possible.

A stationary Tekran speciation system was installed at the Vodarna station. A Tekran 2537B coupled with Tekran 1130 and 1135 units was used for continuous measurements of different atmospheric Hg species (Hg^0^, Hg^II^, and Hg-p). Even though three different species were measured, only the Hg^0^ data were analyzed for this study. The Tekran 2537B detector was calibrated using the built-in permeation source. Recalibration was set to every 24 h, occurring near midnight. To cross-check the internal permeation source, manual calibration was performed by injecting a known amount of Hg^0^ from the Tekran 2505 bell-jar. The injected amount of Hg was calculated using the Dumarey equation (Dumarey et al. 2010). The sampling airflow through the system was set to 1 L min^−1^.

Discontinuous active measurements were performed periodically using Lumex RA 915 M portable analyzer which measures Hg^0^. The detector was set to 1-s sampling intervals and was coupled by GPS tracking software using a mobile phone. Measurements were performed 1 day per week for at least 20 min at each sampling site. Background corrections were applied every 10 min. The instrument was calibrated in-house by Lumex.

The chosen in situ lichens species were *Punctelia subrudecta* and *Flavoparmelia caperata*. They were sampled at the same locations where the transplanted lichens were exposed. For transplanted lichens, *Hypogymnia physodes* was selected as it is one of the lichen species most frequently used in biomonitoring studies (Blum et al. 2012; Horvat et al. 2000). As mentioned before, *Hypoymnia physodes* lichens for transplantation were collected at Pokljuka location since this location is free of anthropogenic influences. The lichens collected for transplantation were packed into nylon net bags (two to three bags) which were placed and exposed on tree branches at a height of 1.5–2 m. The exposure of transplanted lichens lasted for 3 months (detailed deployment plan in the Supplementary Information, Table S1). After collection, bark and other wooden parts were removed from both in situ and transplanted lichens. Clean lichens were lyophilized using the following procedure: 1 min on − 40 °C, vacuum 0.280 mbar; 30 h on 0 °C, vacuum 0.280 mbar; 15 h on 20 °C, vacuum 0.028 mbar; 15 h on 30 °C, vacuum 0.028 mbar; and 10 h final drying at 30 °C, vacuum 0.028 (Christ; Martin Christ Gefriertrocknungsanlagen, Germany). Following the lyophilisation, lichens were ground and homogenized after immersion in liquid nitrogen. The lichens were then analyzed for total Hg content using the digestion method for the determination of Hg in inorganic matrices (Ogrinc et al. 2007). Such digestion was used because of the possibility that the lichens might have adsorbed some inorganic silicon-containing material into their structure during exposure. Approximately 0.2 g of dry sample was weighed in Teflon vials (25 mL); 1 mL of Milli-Q water, 5 mL of a 2:1 (*v*/*v*) HNO_3_/HF mixture, and 1 mL of HCl were slowly added to the sample. The vessels were then capped and left to stand for at least 1 h at room temperature. Teflon vessels were heated at 100 °C and left overnight for digestion. After heating, the samples were cooled to room temperature before opening the vessels. The content of the Teflon vessels was diluted with 5% H_3_BO_3_ solution (*w*/*v*) up to 50 mL. Prior analysis, duplicate reagent blanks and triplicate reference materials were prepared for each analysis batch. The selected certified reference material for quality control was IAEA-336 (lichens), which has a certified total Hg concentration of 0.2 µg g^−1^ dry weight. The concentration of Hg in digested samples was determined using an atomic absorbance spectrometer – Model Hg-201 Semi-automated Mercury Analyzer (Sanso Seisakusho Co. Ltd., Tokyo, Japan).

As regards CNR-PASs, these devices work on the principle of axial diffusion of Hg to a fibrous quartz membrane coated with sorbent material. The filter is attached to the bottom of a borosilicate glass vessel, which is equipped with a double cap system to minimize operator handling and avoid contamination from the cap opening. The sorbent material is made of an aggregation of densely packed TiO_2_ nanoparticles finely decorated with smaller gold nanoparticles (Macagnano et al. 2018). CNR-PASs were deployed in triplicate at three sampling sites for 3, 6, or 12 weeks (detailed deployment plan in the Supplementary Information, Table S2). Furthermore, two CNR-PASs were used as field blanks to check for Hg contamination during deployment. After the desired exposure period, the sorbent membranes were analyzed by thermal decomposition-atomic absorption spectroscopy (TD-AAS) using a Nippon MA-3000 Mercury Analysis System, according to the Environmental Protection Agency (EPA) method 7473 (U.S. EPA 1998). The analysis involved a vaporization step at 220 °C and a decomposition step at 850 °C, followed by gold amalgamation and detection by CV-AAS. External calibration was performed by analyzing five different standard solutions, providing Hg in the range of 0.5–100 ng. Calibration and quality control was carried out with the same protocol using working solution prepared from a standard reference material (NIST SRM 3133 Mercury standard solution).

Passive samplers MerPAS® were used to determine gaseous mercury concentrations. The full protocols for sampling and analysis are described in McLagan et al. (2016). Briefly, the MerPAS® uses radial diffusion of Hg toward a mesh cylinder that contains sulfur-impregnated carbon derived from bituminous coal as a sorbent. The sampling rate is constrained by a microporous polyethylene diffusive barrier (McLagan et al. 2016). Analyses of the samples were conducted using TD-AAS using the Milestone DMA-80 analyzer. MerPAS® was deployed at three sampling sites for 12 weeks (detailed deployment plan in the Supplementary Information, Table S2). The average Hg concentration in the atmosphere measured by each PAS (*C*; ng m^−3^) was obtained from the analyzed mass of Hg in the sorbent material according to Eq. (1)

1 $${\text{C}} \, = \, \frac{\text{m}}{{\text{t}} \, \times \, {\text{SR}}}$$

where *m* is the mass of sorbed Hg (ng) corrected for the concentration in blank samples, *t* is the deployment time of the PAS (days, “d”), and *SR* is the sampling rate of the PAS (m^3^ d^−1^). Experimentally derived *SR* values were used for each PAS. For the CNR-PASs, the *SR* was 0.0147 m^3^ d^−1^ with an uncertainty of 0.0007 m^3^ d^−1^, calculated according to Naccarato et al. (2021). For the MerPASs, the *SR* was 0.131 m^3^ d^−1^ with an uncertainty of 0.003 m^3^ d^−1^, calculated according to McLagan et al. (2018b)

## Results and discussion

### Feasibility of lichens and passive samplers for atmospheric Hg monitoring

To demonstrate the feasibility of selected lichens and PASs for atmospheric Hg monitoring, we compared these monitoring methods with active measurements.

**Fig. 2:**
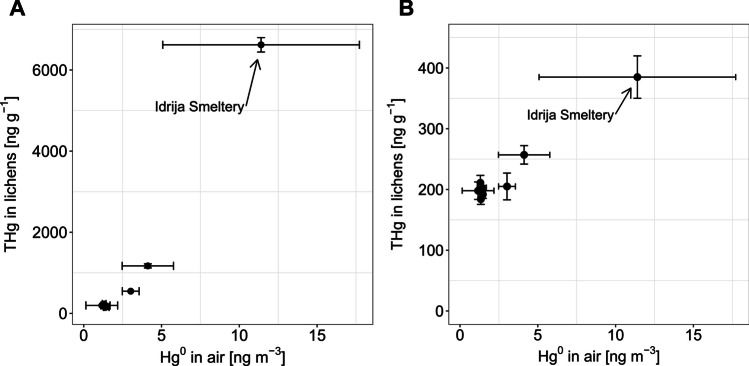
Demonstration of feasibility for monitoring atmospheric Hg using **A** in situ lichens and **B** 3-month transplanted lichens. For Hg^0^ in air (Lumex), whiskers represent the standard deviation of the geometric means of periodic 10-min measurements. For THg in lichens (in situ and 3-month transplanted), whiskers represent the standard deviation of all measured lichens from the same station

For lichens, we compared the concentration of Hg^0^ in air measured by discontinuous active measurements (Lumex) with the total mercury (THg) concentration in lichens (Fig. 2). The data used to construct the figure are shown in the Supplementary Information (Tables S1, S3, and S4). Although Hg^0^ data are often represented by arithmetic means in related studies (McLagan et al. 2018a; Naccarato et al. 2021), our Hg^0^ data are represented by geometric rather than arithmetic means as our dataset followed a geometric rather than normal distribution due to occasional spikes in measured Hg^0^ concentration.

As can be seen in Fig. 2, the variability of Hg^0^ in the air measured by discontinuous measurements is large, especially for the highest concentration point (Idrija Smeltery). The variability originates from the characteristics of the Idrija location (former Hg mine), where Hg concentrations can rise upwards of 100 ng m^−3^ in short periods. Since the in situ lichens were present on the sampling locations for a much longer period than the transplanted lichens, the in situ lichens accumulated Hg for a longer time than transplanted lichens, resulting in the high THg concentrations in in situ. In situ lichens in our study had higher THg concentrations than most studies that measured THg in lichens. This is most evident for in situ lichens in Idrija, where THg concentrations ranged from 547 to 6620 ng g^−1^ (Table S3) compared to literature values that rarely exceed 500 ng g^−1^ (Bargagli 2016). Although all three Idrija stations were located in the general area of the former Hg mine, the highest lichen THg concentration (6620 ng g^−1^) was observed at Idrija Smeltery due to its close proximity to the historical Hg smelting complex, where Hg^0^ concentrations in air are the highest (Kocman et al. 2011). The deployment periods for 3-month transplanted lichens and Lumex measurements did not overlap (Table 1), which could limit the interpretation of the comparison in Fig. 2B. Nevertheless, it is evident that both in situ and transplanted lichens are responsive to elevated atmospheric Hg concentrations. The feasibility of lichens for identifying locations with different atmospheric Hg concentrations was also demonstrated by (Božič et al. 2022) for the same sampling campaign (Božič et al. 2022); therefore, we will not discuss this matter in detail in the present article.

**Fig. 3:**
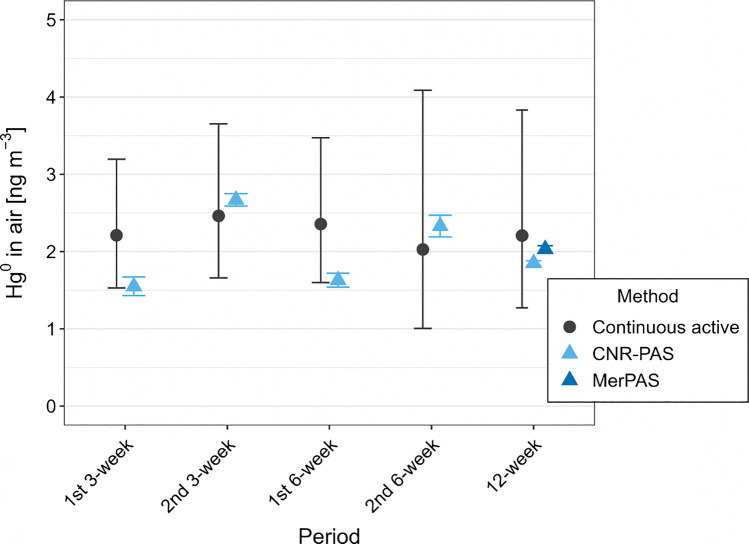
Demonstration of the feasibility of monitoring atmospheric Hg using PASs. For continuous active measurements (Tekran), whiskers represent 95% confidence intervals obtained from geometric means and geometric standard deviations for all measurements during the period. For PASs (CNR-PAS and MerPAS*®*), whiskers represent the standard deviation obtained from three PASs

For PASs, we decided to only use the data from Vodarna station to demonstrate feasibility. The decision was made because the PASs were exposed for shorter periods than the lichens; thus, only time periods with overlap between the deployment of continuous active measurements and PASs were used. This was only possible at the Vodarna station, where continuous active measurements were performed with a stationary Tekran unit. Figure 3 shows the comparison of atmospheric Hg^0^ concentration as determined by PASs and continuous measurements during the same periods. The data used to construct the figure are available in Supplementary Information (Tables S2 and S5).

Figure 3 illustrates that the results of continuous active measurements and PASs at the Vodarna station were in good agreement throughout most periods, the biggest difference was observed for the first 3-week period and the first 6-week period. Ideally, the comparison of the datasets obtained by PASs and continuous active measurements should be performed using measurement uncertainties; however, we did not obtain all data required for a thorough evaluation of measurement uncertainty of all three methods (out of scope of this article). Additionally, the literature does not provide uncertainty values of the methods that could be adopted for our work. The large whiskers for continuous active measurement data are due to characteristic large sample variability at the Anhovo sampling location, while the whiskers for PASs represent replicate reproducibility. Additionally, the datasets do not follow the same distribution (geometric vs. normal). Due to the abovementioned reasons, statistical significance tests could not be performed. Although the dataset characteristics limit the comparison, the results in Fig. 3 suggest that PASs could be a useful tool for monitoring long-term atmospheric Hg concentrations.

### Comparison of lichens and passive samplers

**Fig. 4:**
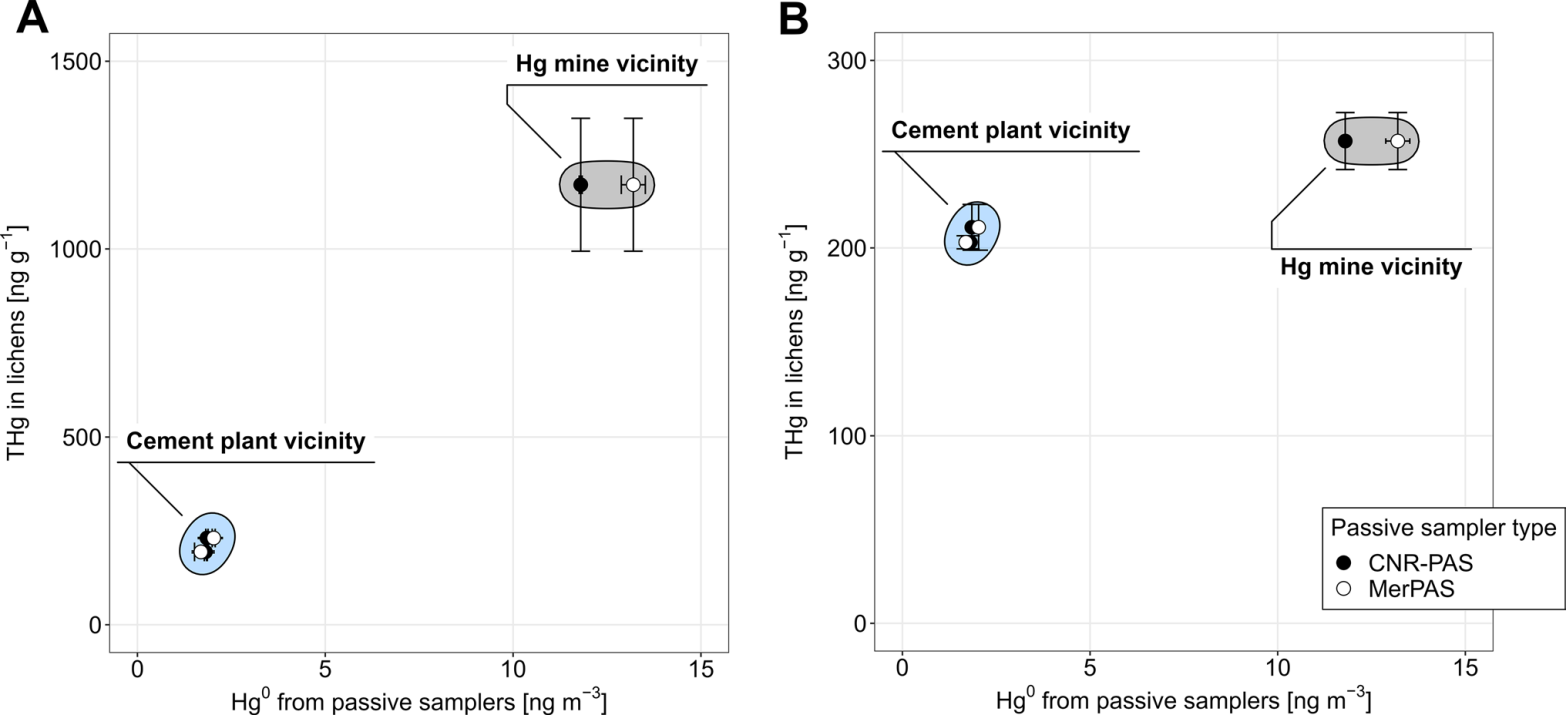
**A** Comparison of in situ lichens and PASs. **B** Comparison of 3-month transplanted lichens and PASs. For THg in lichens (in situ and 3-month transplanted), whiskers represent the standard deviation of all measured lichens from one station. For PASs (CNR-PAS and MerPAS®), whiskers represent the standard deviation obtained from three PASs

Since lichens and passive samplers both proved to be feasible for atmospheric Hg monitoring, we then continued with the comparison of the responses of lichens and PASs to different atmospheric Hg^0^ concentrations at Vodarna, Anhovo, and Idrija Town stations. These stations were selected because PASs were exposed only at these stations. To ensure comparability of results obtained over similar periods, 12-week PASs (both CNR-PAS and MerPAS®) were chosen for this comparison. Twelve-week PAS exposure periods were chosen since they were the most similar to the 3-month periods used for transplanted lichens (though not exactly the same). The results of the comparison are shown in Fig. 4. The data used to create the figure are available in Supplementary Information (Tables S1, S2, and S3).

The obtained comparisons showed that THg concentration in lichens and Hg^0^ concentration from PASs can be efficiently used for identifying areas with distinct Hg emission characteristics. There is an evident grouping of the results obtained from sampling stations in the vicinity of the cement plant (Vodarna and Anhovo stations) and the sampling station in the vicinity of the former Hg mine (Idrija Town). Since transplanted lichens (Fig. 4B) were less responsive to atmospheric Hg concentrations than in situ lichens (Fig. 4A), it is not clear whether areas with smaller differences in atmospheric Hg concentrations than the areas used in our work can be identified with transplanted lichens. Nonetheless, the combination of in situ lichens and PASs and the grouping of the results indicates that areas with elevated atmospheric Hg concentrations can be identified by lichens and PASs. Such identification is more cost-effective and demands less workload than active atmospheric Hg measurements. It can potentially be used for large-scale and long-term monitoring of atmospheric Hg concentrations. Since results for long-term atmospheric Hg concentrations obtained with PASs can sometimes be uncertain (Huang et al. 2014; Huang and Gustin 2015), lichens provide additional independent data that increase the confidence in the obtained results, as demonstrated by our results. This is true even though lichens (as used in our work) cannot provide long-term average atmospheric Hg concentrations in units of ng m^−3^.

### Comparison of discontinuous active measurements and passive samplers

Due to the portability and direct measurements of atmospheric Hg^0^, discontinuous active measurements with the Lumex analyzer can be used as a sensor method for atmospheric Hg concentrations (Pandey et al. 2011). In the following comparison, our goal was to assess whether the results obtained from periodic measurements with the portable analyzer are suitable for long-term monitoring of atmospheric Hg concentrations. Since PASs performed well in our previous tests, we used them as our control to assess the feasibility of atmospheric Hg monitoring with discontinuous active measurements. The relative difference between Hg^0^ concentration as measured by discontinuous active measurements and PASs was used as a measure of feasibility. The relative difference was calculated from the data available in the Supplementary Information (Table S6). The comparison results are shown in Table 2.

**Table 2:** Comparison of discontinuous active measurements and PASs as methods for monitoring long-term atmospheric Hg concentrations

Station	PAS type	Relative difference (Hg^0^_discontinous active_ − Hg^0^_PAS_)/Hg^0^_discontinuous active_
Vodarna	CNR-PAS 3-week	− 87%
	CNR-PAS 6-week	− 51%
	CNR-PAS 12-week	− 42%
	MerPAS® 12-week	− 47%
Anhovo	CNR-PAS 3-week	− 91%
	CNR-PAS 6-week	− 36%
	CNR-PAS 12-week	− 34%
	MerPAS® 12-week	− 21%
Idrija Town	CNR-PAS 3-week	− 281%
	CNR-PAS 6-week	− 283%
	CNR-PAS 12-week	− 281%
	MerPAS® 12-week	− 267%

The results in Table 2 show that in all cases the discontinuous active measurements of Hg^0^ were lower than Hg^0^ as determined from PASs. The difference can be explained by the characteristics of the sampling stations. All stations are located in Hg-polluted areas, the pollution originating from the vicinity of the cement plant (Vodarna and Anhovo) and the former Hg mine (Idrija Town). The proximity of Hg sources causes spikes in the atmospheric Hg^0^ concentration, which are often short-term. Short-term spikes can easily be missed with occasional active measurements, so the concentration obtained by such measurements is lower than the concentration obtained by PASs. PASs are continuously exposed for longer periods (3, 6, and 12 weeks) and capture spikes in Hg^0^ concentration more consistently than discontinuous active measurements. This occurrence is most evident for the Idrija Town station which is known for the high variability of Hg^0^ concentration because of the vicinity of the Hg mine (Kocman et al. 2011; Kotnik et al. 2005). At Idrija Town, the average Hg^0^ concentration from discontinuous active measurements was almost 3 times lower than the Hg^0^ concentration from PASs. The discrepancy could also be explained by the fact that discontinuous measurements are usually not made in a randomized fashion, but typically happen during regular working hours and rarely at night or on weekends (which is also the case in our study), and are therefore inherently biased. Another hypothesis for the observed difference could be that the PASs give biased high results in areas with high Hg^0^ concentration such as Idrija, though the matching results between different PAS types make this hypothesis unlikely. These results indicate that discontinuous active measurements do not allow adequate exposure assessment because of the tendency to underestimate peak concentrations, especially near point sources of Hg pollution. More frequent active measurements and better overlap between the PAS exposure time and discontinuous active measurements would probably improve results and comparability, but at the cost of using fewer sampling stations and a higher workload. The results in Table 2 also imply that the values of Hg^0^ concentrations in air in Fig. 2 are underestimated, since these values were obtained with discontinuous active measurements. This means that the *x*-axis values in Fig. 2 should be shifted towards higher values, especially the highest concentration points (i.e., Idrija Smeltery). Nevertheless, this shift does not affect the assessment of lichen feasibility, as the responsiveness of lichens to elevated atmospheric Hg concentrations would still be evident.

## Conclusions

The combined use of passive sampling and biomonitoring with lichens was shown to be a strong alternative to active measurements for identification of locations with elevated Hg concentrations. However, since locations with very different atmospheric Hg concentrations were used, the feasibility of the aforementioned methods cannot be generalized to distinguish differences between locations at background atmospheric Hg concentrations based on our work. Variable atmospheric Hg concentrations were better captured by lichens and PASs due to long exposure times (several weeks) in comparison to short (10-min) discontinuous active measurements. The reasons for the disagreement between discontinuous active measurements and PASs need further exploration; long-term averages of discontinuous measurements could be improved by more frequent measurements and a randomized design that includes measurements at night and during weekends. Our work shows that biomonitoring and PASs can offer a cost-effective alternative to continuous active measurements, if the goal is long-term atmospheric Hg monitoring and identification of areas with elevated atmospheric Hg concentrations. Nonetheless, continuous active methods still provide better temporal resolution and offer measurements of additional atmospheric Hg species (Hg^II^ and Hg-p) in comparison to biomonitoring and passive sampling.

### Supplementary Information

Below is the link to the electronic supplementary material.

Supplementary file1 (DOCX 38 KB)

## Data Availability

Data available within the article or its supplementary materials.
