# Supplementary material for: Comparison of active measurements, lichen biomonitoring, and passive sampling for atmospheric mercury monitoring
Source: Environ Sci Pollut Res Int. 2024 May 14;31(24):35800–10. doi: 10.1007/s11356-024-33582-6 (PMC11136809; doi:10.1007/s11356-024-33582-6)
Supplement: Supplementary file 1 — Supplementary file1 (DOCX 38 KB) [file 11356_2024_33582_MOESM1_ESM.docx]

Supplementary Information for:

Comparison of Active Measurements, Lichen Biomonitoring, and Passive Sampling for Atmospheric Mercury Monitoring

Jan Gačnik^1,2^, Igor Živković^1,2^, Jože Kotnik^1,2^, Dominik Božič^1,2^, Antonella Tassone^3^, Attilio Naccarato^3,4^, Nicola Pirrone^3^, Francesca Sprovieri^3^, Alexandra Steffen^5^ and Milena Horvat^1,2#^

^1^Department of Environmental Sciences, Jožef Stefan Institute, Ljubljana, Slovenia

^2^Jožef Stefan International Postgraduate School, Ljubljana, Slovenia

^3^Institute of Atmospheric Pollution Research, National Research Council, Rende, Italy

^4^Department of Chemistry and Chemical Technologies, University of Calabria, Rende, Italy

^5^Air Quality Research Division, Environment and Climate Change Canada, Toronto, Canada

^#^Corresponding author, available at: milena.horvat@ijs.si

Table S1 Sampling start and end date with obtained results for the exposure of 3-months transplanted lichens. Columns “Bag 1”, “Bag 2”, and “Bag 3” indicate values obtained for transplanted lichens contained in the same exposure bag

| Sampling site | Coordinates (°N, °E) | Start date | End date | Bag 1  THg [ng g^−1^] | Bag 2  THg [ng g^−1^] | Bag 3  THg [ng g^−1^] | Average (SD)  [ng g^−1^] |
| --- | --- | --- | --- | --- | --- | --- | --- |
| Vodarna | 46.06243, 13.61903 | 16/01/2020 11:30 | 6/5/2020 10:30 | 214 | 200 | 220 | 211 (12.2) |
| Anhovo | 46.05718, 13.61727 | 17/01/2020 12:30 | 6/5/2020 11:30 | 205 | 201 | / | 203 (3.49) |
| Morsko | 46.07164, 13.6323 | 17/01/2020 13:30 | 6/5/2020 11:50 | 193 | 190 | / | 192 (6.71) |
| Ročinj | 46.10869, 13.68137 | 30/01/2020 11:30 | 6/5/2020 12:10 | 189 | 179 | / | 184 (8.55) |
| Spodnja Idrija | 46.0733, 14.02455 | 23/01/2020 14:30 | 6/5/2020 13:10 | 183 | 202 | 230 | 205 (22.0) |
| Idrija Town | 46.00211, 14.02451 | 23/01/2020 15:00 | 6/5/2020 13:20 | 246 | 265 | 260 | 257 (15.2) |
| Idrija Smeltery | 46.00762, 14.03137 | 23/01/2020 15:00 | 6/5/2020 13:30 | 416 | 350 | 392 | 285 (34.9) |
| Pokljuka | 46.34536, 13.95298 | 08/03/2020 11:00 | 9/6/2020 11:00 | 193 | 214 | 187 | 198 (14.4) |

Table S2 Sampling start and end date with obtained results for the exposure of passive samplers. Averages and standard deviations were obtained from three exposed passive samplers during the same time period at the same sampling site

| Sampling site | Passive sampler | Campaign | Start date | End date | Average air Hg^0^ conc. (SD) [ng m^−3^] |
| --- | --- | --- | --- | --- | --- |
| Vodarna | CNR 12-week | First campaign | 21/02/2020 10:50 | 13/05/2020 10:30 | 1.85 (0.03) |
|  | CNR 1^st^ 6-week |  | 17/02/2020 11:30 | 01/04/2020 11:00 | 1.63 (0.09) |
|  | CNR 2^nd^ 6-week |  | 01/04/2020 11:00 | 13/05/2020 10:30 | 2.33 (0.14) |
|  | CNR 1^st^ 3-week |  | 17/02/2020 11:30 | 10/03/2020 11:30 | 1.55 (0.12) |
|  | CNR 2^nd^ 3-week |  | 10/03/2020 11:30 | 01/04/2020 11:00 | 2.67 (0.08) |
|  | Mer-PAS 12-week |  | 17/02/2020 11:30 | 18/05/2020 10:00 | 2.03 (0.04) |
|  | CNR 12-week | Second campaign | 16/07/2020 10:30 | 16/10/2020 11:00 | 1.86 (0.03) |
|  | CNR 1^st^ 6-week |  | 16/07/2020 10:30 | 07/09/2020 11:00 | 1.70 (0.58) |
|  | CNR 2^nd^ 6-week |  | 07/09/2020 11:00 | 16/10/2020 11:00 | 2.27 (0.19) |
|  | CNR 1^st^ 3-week |  | 16/07/2020 10:30 | 14/08/2020 11:00 | 4.24 (0.09) |
|  | CNR 2^nd^ 3-week |  | 14/08/2020 11:00 | 07/09/2020 11:00 | 1.35 (0.19) |
|  | CNR 3^rd^ 3-week |  | 07/09/2020 11:00 | 28/09/2020 11:00 | 3.93 (0.75) |
|  | CNR 4^th^ 3-week |  | 28/09/2020 11:00 | 16/10/2020 11:00 | 0.96 (0.53) |
|  | Mer-PAS 12-week |  | 18/05/2020 10:00 | 14/08/2020 10:30 | 1.83 (0.14) |
| Anhovo | CNR 12-week | First campaign | 21/02/2020 12:00 | 13/05/2020 10:30 | 1.81 (0.03) |
|  | CNR 1^st^ 6-week |  | 17/02/2020 13:30 | 01/04/2020 12:00 | 1.78 (0.09) |
|  | CNR 2^nd^ 6-week |  | 01/04/2020 12:00 | 13/05/2020 10:30 | 1.30 (0.06) |
|  | CNR 1^st^ 3-week |  | 17/02/2020 13:30 | 10/03/2020 12:30 | 1.86 (0.21) |
|  | CNR 2^nd^ 3-week |  | 10/03/2020 12:30 | 01/04/2020 12:00 | 2.41 (0.11) |
|  | Mer-PAS 12-week |  | 17/02/2020 13:30 | 18/05/2020 11:00 | 1.69 (0.17) |
|  | CNR 12-week | Second campaign | 16/07/2020 11:30 | 16/10/2020 12:00 | 1.95 (0.05) |
|  | CNR 1^st^ 6-week |  | 16/07/2020 11:30 | 07/09/2020 12:00 | 1.89 (0.38) |
|  | CNR 2^nd^ 6-week |  | 07/09/2020 12:00 | 16/10/2020 12:00 | 2.66 (0.21) |
|  | CNR 1^st^ 3-week |  | 16/07/2020 11:30 | 14/08/2020 12:00 | 3.55 (0.29) |
|  | CNR 2^nd^ 3-week |  | 14/08/2020 12:00 | 07/09/2020 12:00 | 2.41 (0.20) |
|  | CNR 3^rd^ 3-week |  | 07/09/2020 12:00 | 28/09/2020 12:00 | 3.75 (0.03) |
|  | CNR 4^th^ 3-week |  | 28/09/2020 12:00 | 16/10/2020 12:00 | 2.04 (0.22) |
|  | Mer-PAS 12-week |  | 18/05/2020 11:00 | 14/08/2020 11:30 | 1.70 (0.10) |
| Idrija Town | CNR 12-week | First campaign | 21/02/2020 13:20 | 13/05/2020 10:30 | 11.8 (0.04) |
|  | CNR 1^st^ 6-week |  | 17/02/2020 17:30 | 01/04/2020 14:00 | 9.38 (0.03) |
|  | CNR 2^nd^ 6-week |  | 01/04/2020 14:00 | 13/05/2020 10:30 | 15.1 (0.08) |
|  | CNR 1^st^ 3-week |  | 17/02/2020 17:30 | 10/03/2020 14:30 | 12.5 (0.07) |
|  | CNR 2^nd^ 3-week |  | 10/03/2020 14:30 | 01/04/2020 14:00 | 7.90 (0.02) |
|  | Mer-PAS 12-week |  | 17/02/2020 17:30 | 18/05/2020 13:00 | 13.2 (0.33) |
|  | CNR 12-week | Second campaign | 16/07/2020 13:30 | 16/10/2020 14:00 | 19.6 (0.21) |
|  | CNR 1^st^ 6-week |  | 16/07/2020 13:30 | 07/09/2020 14:00 | 24.5 (0.06) |
|  | CNR 2^nd^ 6-week |  | 07/09/2020 14:00 | 16/10/2020 14:00 | 14.2 (0.04) |
|  | CNR 1^st^ 3-week |  | 16/07/2020 13:30 | 14/08/2020 14:00 | 25.4 (0.08) |
|  | CNR 2^nd^ 3-week |  | 14/08/2020 14:00 | 07/09/2020 14:00 | 19.3 (0.07) |
|  | CNR 3^rd^ 3-week |  | 07/09/2020 14:00 | 28/09/2020 14:00 | 19.7 (0.06) |
|  | CNR 4^th^ 3-week |  | 28/09/2020 14:00 | 16/10/2020 14:00 | 9.28 (0.11) |
|  | Mer-PAS 12-week |  | 18/05/2020 13:00 | 14/08/2020 13:30 | 17.0 (1.40) |

Table S3 Collection date and obtained results for the in-situ lichens. THg represents the average results obtained from two digestions of the same sample

| Sampling site | Collection date | THg [ng g^−1^] |
| --- | --- | --- |
| Vodarna | 16/1/2020 11:30 | 231 |
| Anhovo | 17/1/2020 12:30 | 194 |
| Morsko | 17/1/2020 13:30 | 159 |
| Ročinj | 30/1/2020 13:00 | 162 |
| Spodnja Idrija | 23/1/2020 14:30 | 547 |
| Idrija Town | 23/1/2020 15:00 | 6620 |
| Idrija Smeltery | 23/1/2020 15:00 | 1171 |
| Pokljuka | 16/12/2019 13:00 | 174 |

Table S4 Measurement date and obtained results for the discontinuous active measurements using Lumex portable analyzer. 10-minute measurements were performed on each measurement date. Geometric mean was used as the estimator due to the distribution of individual measurement points during the 10-minute measurement interval

| Sampling site | Date of measurement | Geomean (GSD) [ng m^−3^] | Mean of geomeans (SD) [ng m^−3^] |
| --- | --- | --- | --- |
| Vodarna | 16/10/2020 | 1.20 (2.08) | 1.31 (0.12) |
|  | 06/11/2020 | 1.49 (2.13) |  |
|  | 11/11/2020 | 1.29 (1.98) |  |
|  | 29/10/2020 | 1.27 (2.10) |  |
| Anhovo | 16/10/2020 | 1.81 (1.94) | 1.40 (0.29) |
|  | 06/11/2020 | 1.21 (2.15) |  |
|  | 11/11/2020 | 1.20 (2.16) |  |
|  | 29/10/2020 | 1.38 (2.09) |  |
| Morsko | 16/10/2020 | 1.69 (1.98) | 1.47 (0.16) |
|  | 06/11/2020 | 1.33 (2.12) |  |
|  | 11/11/2020 | 1.47 (2.01) |  |
|  | 29/10/2020 | 1.40 (2.07) |  |
| Ročinj | 16/10/2020 | 1.27 (2.07) | 1.34 (0.08) |
|  | 06/11/2020 | 1.29 (2.13) |  |
|  | 11/11/2020 | 1.45 (1.91) |  |
|  | 29/10/2020 | 1.35 (2.11) |  |
| Spodnja Idrija | 16/10/2020 | 2.56 (1.85) | 3.02 (0.54) |
|  | 06/11/2020 | 2.82 (1.82) |  |
|  | 11/11/2020 | 2.89 (1.62) |  |
|  | 29/10/2020 | 3.79 (1.71) |  |
| Idrija Town | 16/10/2020 | 5.91 (3.08) | 4.12 (1.65) |
|  | 06/11/2020 | 2.07 (2.28) |  |
|  | 11/11/2020 | 3.66 (1.86) |  |
|  | 29/10/2020 | 4.85 (2.03) |  |
| Idrija Smeltery | 16/10/2020 | 13.9 (5.12) | 11.4 (6.32) |
|  | 06/11/2020 | 3.39 (2.61) |  |
|  | 11/11/2020 | 18.3 (5.23) |  |
|  | 29/10/2020 | 10.1 (2.30) |  |
| Pokljuka | 15/12/2020 | 1.16 (N/A) | / |

Table S5 Results obtained from continuous active measurements at Vodarna station which were used for comparison with passive samplers. We used continuous active measurement data obtained in the same period to the period when individual passive samplers were exposed to ensure best comparability.

| Time period | Passive sampler exposed in that period | Geomean Hg^0^ conc. from Tekran (GSD) |
| --- | --- | --- |
| 17/02/2020 – 10/03/2020 | CNR 1st 3-week | 2.21 (1.45) |
| 10/03/2020 – 1/04/2020 | CNR 2nd 3-week | 2.46 (1.48) |
| 17/02/2020 – 1/04/2020 | CNR 1st 6-week | 2.36 (1.47) |
| 1/04/2020 – 13/05/2020 | CNR 2nd 6-week | 2.03 (2.02) |
| 17/02/2020 – 13/05/2020 | CNR 12-week  and Mer-PAS 12-week | 2.21 (1.74) |

Table S6 Full results of the comparison of discontinuous active measurements and passive samplers as methods for monitoring long-term atmospheric Hg concentrations. Values in the second column were obtained from Table S4 while values in fourth column were obtained by averaging all data for individual passive sampler types from Table S2

| Sampling site | Average Hg^0^ conc. discontinuous active (SD) [ng m^−3^] | Passive sampler | Average Hg^0^ conc. passive sampler (SD) [ng m^−3^] | Relative difference Hg^0^_discontinous active_ − Hg^0^_passive sampler_ |
| --- | --- | --- | --- | --- |
| Vodarna | 1.31 (0.12) | CNR 3-week | 2.45 (0.75) | −87% |
|  |  | CNR 6-week | 1.98 (0.58) | −51% |
|  |  | CNR 12-week | 1.86 (0.05) | −42% |
|  |  | Mer-PAS 12-week | 1.93 (0.15) | −47% |
| Anhovo | 1.40 (0.29) | CNR 3-week | 2.67 (0.29) | −91% |
|  |  | CNR 6-week | 1.91 (0.38) | −36% |
|  |  | CNR 12-week | 1.88 (0.05) | −34% |
|  |  | Mer-PAS 12-week | 1.69 (0.12) | −21% |
| Idrija Town | 4.12 (1.65) | CNR 3-week | 15.7 (0.11) | −281% |
|  |  | CNR 6-week | 15.8 (0.08) | −283% |
|  |  | CNR 12-week | 15.7 (0.21) | −281% |
|  |  | Mer-PAS 12-week | 15.1 (2.28) | −267% |
